# Editorial: Helminthosis: immuno-pathology and anthelmintic vaccines

**DOI:** 10.3389/fimmu.2025.1727852

**Published:** 2025-11-17

**Authors:** Joydeep Paul, Muhammad Tofazzal Hossain, Md. Aminul Islam, Anisuzzaman

**Affiliations:** 1 Department of Biotechnology, School of Life Science and Biotechnology, Adamas University, Kolkata, India; 2 Department of Microbiology and Hygiene, Bangladesh Agricultural University, Mymensingh, Bangladesh; 3 Department of Medicine, Bangladesh Agricultural University, Mymensingh, Bangladesh; 4 Department of Parasitology, Bangladesh Agricultural University, Mymensingh, Bangladesh

**Keywords:** helminthosis, immunopathology, anthelmintic vaccines, extracellular vesicles (EVs), host–parasite interaction, immune modulation

**Figure 1:**
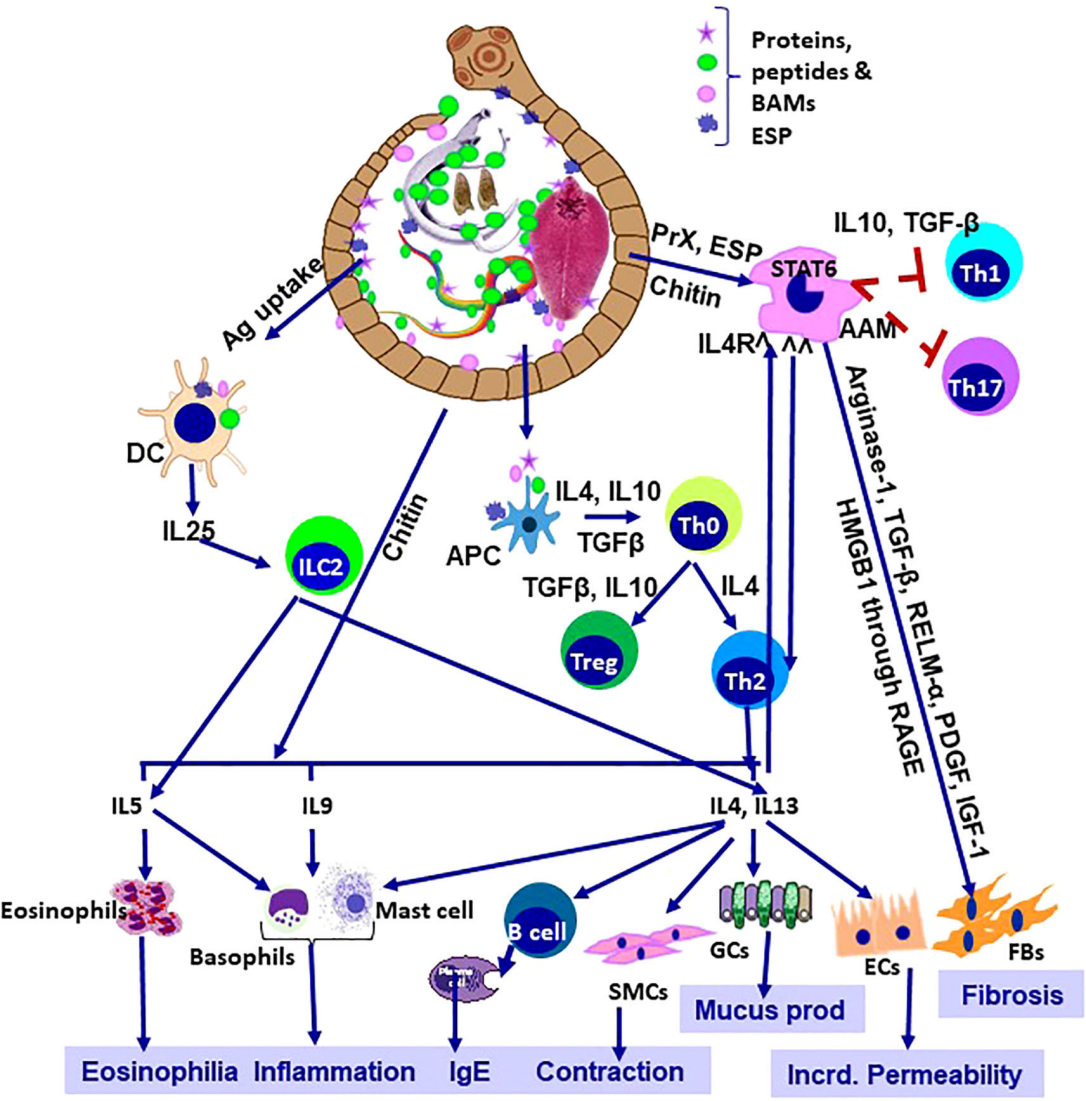
Helminthiosis: Immunological orchestra and patho-biology. AAM, alternatively activated macrophage; APC, antigen presenting cell; DC, dendritic cell; SMCs, smooth muscle cells, GCs, goblet cells; Ecs, epithelial cells; FBs, fibroblasts; Incrd, increased; Ag, antigens; BAMs, bioactive molecules, PrX, 2-Cys peroxiredoxin; RELM-α, Resistin-like Molecule- α; PDGF, platelet-derived growth factor; IGF-1, insulin-like growth factor 1; HMGB1, High-Mobility Group Box 1, RAGE, receptor for advanced gycation end-products; ESP, excretory-secretory products.

Helminth infections remain a major global challenge, especially in the tropics and the sub-tropics. While they rarely receive the attention accorded to tuberculosis or malaria, their burden is considerable, resulting in growth impairment, infertility, chronic disability, and premature mortality (1–3). Co-evolution of these helminths along with their respective hosts is the key to their survival and persistence, even in the modern world (4). Initially, helminth infections induce a Th1 immune response, accompanied by increased IgE and T cell activation. Later in the chronic illness, polarization of the host immune response towards Th2 helps the parasites to survive in a more favourable environment (5). The immune response to the helminths is inherently paradoxical. Protective in some contexts, yet can be manipulated by the parasites to ensure survival (**Figure 1**). This duality is central to the immunopathology examined in this Research Topic. Helminths also actively induce Treg cells, which helps the helminth’s survival by suppressing the host’s immune response. Helminth-induced Treg ameliorates “bystander” immune responses, protecting against allergies and autoimmune diseases (6). The pursuit of vaccines is equally important, which, despite decades of work, lags behind efforts against bacterial and viral diseases (7). This study offered fresh perspectives on these intertwined challenges. This Research Topic will also help us to understand the host-parasite interactions more intricately by highlighting the underlying molecular mechanisms, particularly in the field of vaccine development.

The issue discussed many newer and evolving problems related to helminthosis. Ten different articles range from giving intricate highlights in understanding the modulation of immune responses and other host inflammatory responses in helminthosis to the development of protective responses against different helminths, either through regular physical exercises or with the advancement of new prophylactic and therapeutic modules. Sternkopf et al. showed that the vesicular fluid derived from the *Taenia soliu*m cyst (CVF) induces Caspase 3 and 9 mediated apoptosis in human neurocysticercosis patients by significantly inhibiting mitochondrial function. This article also identifies that elevated FasL levels act as a key mediator to induce brain inflammation by primarily targeting monocytes, microglia, and in CD3+ expressing T cells. Yuan et al. demonstrated how *Nippostrongylus brasiliensis*-derived uridine alleviates colitis in mice via apical sodium-dependent bile acid transporter (ASBT). Their findings suggest novel molecular targets for treating inflammatory bowel diseases and also suggest how helminth biology can reform wider field of medicine. Schulze et al. characterize the immune remodelling in *Schistosoma mansoni* infection by documenting granulocyte expansion, cytokine dynamics and the interplay between Th1 and Th2 responses. They contribute significantly to understanding the late-stage disease and its systemic immunological impact. Adjah et al. broaden our knowledge on compartmentalized immunity by showing that portal lymph nodes, along with the mesenteric nodes, generate parasite-specific follicular helper T and B cells during *Heligmosomoides bakeri* infection. Lema et al. analyze immune responses in HIV-positive individuals with *Taenia solium* cysticercosis. Their observations show cytokine patterns and CD4 variations in a sex-biased manner, having stronger responses in males. This study also underscores the complexity of co-infections and the need for tailored management strategies. Lopez et al. reviewed how ILC2 activity correlates with egg burden, IgE production and tryptase levels in *Ascaris lumbricoides* infection, thus linking innate immune responses with allergic and inflammatory consequences. While Sanku et al. emphasized the impaired APC activity and the suppression of the T cell responses by a filarial nematode (*Brugia malayi*) derived EVs. Their work also illustrates the inhibitory role of parasite-derived EVs in cytokine production and may extend to several dissimilar antigens, such as SARS-CoV-2.

Addressing the prophylactic and therapeutic challenges against helminthosis, Liu et al. identified various glycoprotein components of the H11 antigen and also demonstrated that combinations of recombinant cocktails can reproduce a significant amount of protection as seen with native H11 antigen. With barbervax still being considered as the sole commercial helminth vaccine, such advances mark critical progress towards recombinant platforms. Addressing co-endemicity, Zhong et al. reviewed the role of EV-derived *let*-7 microRNAs in shaping host-parasite interactions. Their synthesis highlights both conserved pathways and translational challenges, pointing toward diagnostic and therapeutic opportunities. Lastly, Berkiks et al. contributed in a novel way by showing that moderate exercise reduces neuroinflammation and improves memory in mice infected with schistosomiasis. By linking lifestyle interventions to disease outcomes, this study broadens the scope of the helminth research beyond parasite clearance.

In summary, all ten articles not only provided insights into how helminths use different strategies for their survival in the host, but also suggested diverse approaches that could be used to combat infection. From cellular pathways and EV microRNAs to vaccine antigens and exercise-based interventions, the breadth of inquiry represented here is striking. Importantly, modern understanding unmasked that helminths are not only pathogens but also sources of therapeutic insights, resulting a twist whether helminths are ‘friends or foe’. This Research Topic also highlights the ongoing need for increased visibility in helminth research. Despite their enormous global burden, particularly in low and middle-income countries, these diseases risk neglect amid shifting public health priorities. By bringing together diverse work, this Research Topic highlights both the scientific vibrancy of helminthology and its translational relevance for wider immunological and inflammatory conditions.

In conclusion, this Research Topic is more than a compilation of papers. The studies here remind us that advancing vaccines and therapies against these parasites requires scientific rigor but also collaborative persistence. May it inspire further inquiry and innovation in tackling helminthiosis and its far-reaching consequences.
